# A U-Box Type E3 Ubiquitin Ligase Prp19-Like Protein Negatively Regulates Lipid Accumulation and Cell Size in *Chlamydomonas reinhardtii*

**DOI:** 10.3389/fmicb.2022.860024

**Published:** 2022-04-06

**Authors:** Qiulan Luo, Hui Zhu, Chaogang Wang, Yajun Li, Xianghui Zou, Zhangli Hu

**Affiliations:** ^1^School of Life Sciences and Food Engineering, Hanshan Normal University, Chaozhou, China; ^2^Guangdong Provincial Key Laboratory for Plant Epigenetics, Shenzhen Engineering Laboratory for Marine Algal Biotechnology, Guangdong Technology Research Center for Marine Algal Bioengineering, College of Life Sciences and Oceanography, Longhua Innovation Institute for Biotechnology, Shenzhen University, Shenzhen, China; ^3^Hainan Provincial Key Laboratory for Functional Components Research and Utilization of Marine Bio-Resources, Institute of Tropical Bioscience and Biotechnology, Hainan Academy of Tropical Agricultural Resource, Chinese Academy of Tropical Agricultural Sciences, Haikou, China

**Keywords:** Prp19-like protein, E3 ubiquitin ligase, cell cycle, fatty acids, *Chlamydomonas Reinhardtii*

## Abstract

Microalgae lipid triacylglycerol is considered as a promising feedstock for national production of biofuels. A hotspot issue in the biodiesel study is to increase TAG content and productivity of microalgae. Precursor RNA processing protein (Prp19), which is the core component of eukaryotic RNA splice NTC (nineteen associated complex), plays important roles in the mRNA maturation process in eukaryotic cells, has a variety of functions in cell development, and is even directly involved in the biosynthesis of oil bodies in mouse. Nevertheless, its function in *Chlamydomonas reinhardtii* remains unknown. Here, transcriptional level of *CrPrp19* under nutrition deprivation was analyzed, and both its RNA interference and overexpressed transformants were constructed. The expression level of *CrPrp19* was suppressed by nitrogen or sulfur deficiency. Cell densities of *CrPrp19* RNAi lines decreased, and their neutral lipid contents increased 1.33 and 1.34 times over those of controls. The cells of *CrPrp19* RNAi lines were larger and more resistant to sodium acetate than control. Considerably none of the alterations in growth or neutral lipid contents was found in the *CrPrp19* overexpression transformants than wild type. Fatty acids were also significantly increased in *CrPrp19* RNAi transformants. Subcellular localization and yeast two-hybrid analysis showed that CrPrp19 was a nuclear protein, which might be involved in cell cycle regulation. In conclusion, CrPrp19 protein was necessary for negatively regulating lipid enrichment and cell size, but not stimulatory for lipid storage.

## Introduction

Recently, to achieve the global carbon neutral target, research scientists have put a lot of effort into exploring different sources of green energy, such as biofuels (biodiesel, bioethanol, and bio oil). Among them, biodiesel, which is considered as a high-quality substitute for fossil fuels, has gradually become the research focus for bioenergy science (Ghasemi et al., 2012). As the third-generation biofuels, the biodiesel from microalgae has multiple advantages, i.e., enormous algal species, higher biomass and productivity than oleaginous crops, and short life cycles. Besides, microalgae is able to grow on non-arable land, absorb a large amount of CO_2_, and purify wastewater. Moreover, triacylglycerol (TAG) of microalgae is easy to produce biodiesel through transesterification (Enamala et al., 2018). However, a high production cost has been a big hindrance in the development of biodiesel from microalgae. Since the selection of lipid-rich microalgae is the first step of promoting algae biodiesel production (Wood, 2021), numerous research effort has been done on lipid yield and high biomass-growth algal species breeding. Genetic engineering of target genes, which are involved in lipid biosynthesis pathway, successfully increased TAG contents in microalgae. For example, co-overexpression of two endogenous key enzymes responsible for TAG biosynthesis, plastidial lysophosphatidic acid acyltransferase (LPAAT1) and diacylglycerol acyltransferase 2 (DGAT2), increased neutral lipid contents in *Neochloris oleoabundans* twofold (Chungjatupornchai and Fa-Aroonsawat, 2020). Meanwhile, the knockout or silencing of genes involved in lipid biosynthesis could also improve the yields of natural edible oil and biofuel production (Shin et al., 2018; Nguyen et al., 2020). Nevertheless, the increasing trend of improving TAG synthesis by using genetic engineering techniques were lower than that caused by certain nutrient or element deficiencies. For instance, deficiencies of nitrogen (N) and sulfur (S), which are essential macroelements for the growth of microalgae, enhanced relative contents of TAG in *Chlamydomonas reinhardtii* about 29.1- and 16.5-fold, respectively (Cakmak et al., 2012). Furthermore, it has been revealed that the deficiency of iron (Fe), a microelement, could also increase TAG accumulation in microalgae (Devadasu et al., 2019). However, the lipid generation stimulated by nutrition deprivation was substantially accompanied by serious biomass growth reduction (Breuer et al., 2012). Moreover, the operation of nutrition deficiencies in large-scale production of microalgal biodiesel is difficult. Therefore, it may be necessary to combine nutrient deficiency with genetic engineering. Thus, further investigation of the molecular mechanism of TAG biosynthesis in microalgae is required.

By now, based on the enormous experimental data, the microalgae TAG biosynthesis pathway has been uncovered, which is similar to that in higher plants (Wagner et al., 2010). There are two distinct and parallel TAG biosynthesis pathways in *C. reinhardtii*. One is the Kennedy pathway, using diacylglycerol acyltransferase (DGAT) as the final key rate-limiting enzyme to catalyze diacylglycerol (DAG) acylation, resulting in TAG formation (Russa et al., 2012). In another pathway, TAG is generated from phospholipid and DAG was catalyzed by phospholipid:diacylglycerol acyltransferase (PDAT) (Yoon et al., 2012). Overexpression of *DGAT*s and *PDAT* encoding genes could alter the lipid contents in microalgae, but not all *DGAT* genes affect TAG biosynthesis. When transgenic silencing of five *DGAT*2 genes were carried out, only *CrDGAT2-1* and *CrDGAT2-5* silencing showed lipid content decreases (Deng et al., 2012). Besides, the catabolic enzymes of DAG biosynthesis were also considered as lipid metabolism key enzymes, including glycerol-3-phosphate (G3P), fructose 1,6-bisphosphatase (FBP), phosphoenolpyruvate carboxylase (PEPC), phosphoenolpyruvate carboxykinase (PEPCK), citrate synthase (CIS), and phosphatidic acid phosphatase (PAP) (Moellering et al., 2009). Moreover, phosphofructokinase (PFK), a key regulatory enzyme in glycolysis (Kiss et al., 1989), and phosphoglycolate phosphatase (PGP), which is involved in photorespiration and gluconeogenesis (Suzuki, 1995), both affect lipid metabolism in microalgae from the very beginning. In all, the TAG biosynthesis in microalgae is a very comprehensive metabolism network regulated at transcript, translation, and post-translation levels.

E3 ubiquitin ligases are the last step components in the ubiquitination, which is a vital posttranslational mechanism in regulating many physiological processes, including proteasome degradation, endocytic trafficking, DNA replication, signal transduction, transcription, and apoptosis. U-box E3s conserving U-box motifs play diverse functions in eukaryotes, including lipid metabolism (Mudgil et al., 2004). Precursor mRNA processing factor 19 (Prp19), as the core component of eukaryotic RNA spliceosomal complex NTC (nineteen associated complex), is a typical U-box family member. The U-box domain of Prp19 forms a dimer to maintain the tetrameric protein structure, which functions as an E3 ligase (Kooi et al., 2006). Prp19 protein is not only necessary for activation and structurally stabilizing of NTC spliceosome but also essential for mRNA splicing reaction (Chan et al., 2003). Since Prp19 protein was isolated in yeast through the complementary temperature-sensitive growth defect mutant in 1993 (Cheng et al., 1993), its many roles have been revealed gradually, including regulating cell cycle, DNA repair, maintaining genomic stability, RNA splicing, and others (Grey et al., 1996; Lu and Legerski, 2007). In mammalian cells, other accurate functions of Prp19 proteins were identified with the development of biology, such as inhibiting cell death under hypoxia condition, controlling neurologic tissue differentiation, and participating in neuron and astrocyte anti-aging process (Sato et al., 2010; Yamada et al., 2013).

Previous studies have demonstrated that Prp19 is generally localized in the nucleus. Cho et al. (2007) reported that Prp19 was also localized in lipid droplets of mouse adipocytes, and the knock-down of *Prp19* resulted in reduced triglyceride accumulation (Cho et al., 2007). By far, only one report has shown that Prp19 functions in lipid droplet biogenesis, so the roles of Prp19 in other species still need to be verified. Besides, fewer studies have been done on plant Prp19 proteins than animal Prp19. The plant Prp19 proteins participate in the co-transcriptional assembly of the spliceosome, and crosstalk with DNA repair and cell death signaling pathways (Koncz et al., 2012). Moreover, Prp19 proteins in plant cells are involved in the regulation of plant endogenous immune response (Monaghan et al., 2009), but the detailed functions of plant Prp19 need further exploration. In previous studies, Prp19 in *C. reinhardtii* was one of the most decreased genes in nitrogen deprivation transcriptome. Therefore, to illustrate CrPrp19’s roles in lipid metabolism of *C. reinhardtii*, gene expressions under nutrition deficiencies, gene silencing *via* mRNA interference, gene overexpression, and subcellular localization analysis were conducted. The expression levels of genes encoding lipid biosynthesis–related enzymes were detected in *CrPrp19* RNAi lines. The results will be helpful for understanding the regulatory network of lipid biosynthesis in *C. reinhardtii*, and the excellent transgenic algae obtained could also be a good algal strain resource for future work.

## Materials and Methods

### Algal Strain and Growth Conditions

*C. reinhardtii* CC425 and CC124 were obtained from the College of Life Sciences and Oceanography, Shenzhen University, and grown in tris-acetate-phosphate (TAP) medium at 24°C under continuous illumination (about 120 μmol photons m^–2^ s^–1^) for 3 days to reach a logarithmic phase as of seeds. For nutrition starvation, cells in the middle log stage were inoculated at the ratio of 10% (v/v) into Sueoka’s High-Salt Medium (HSM) or without nitrogen (-N), sulfur (-S), or iron (-Fe) for 8 days. Cultures were collected by centrifugation and used for total RNA isolation. For cell growth, neutral lipid contents, and fatty acid (FA) assays of *CrPrp19* RNAi or overexpression lines, cells were cultured in HSM medium with an initial density of about 5 × 10^5^ cells⋅ml^–1^. For synchronized culture, cells were grown with aeration of 5% (v/v) CO_2_ enriched air on a 14/10 h light/dark cycle for 4 days (Dillard et al., 2011), then cells were collected and observed using an optical microscope.

### Isolation of *CrPrp19* Gene in *Chlamydomonas reinhardtii*

*CrPrp19* complementary DNA (cDNA) sequence was achieved from JGI *Chlamydomonas reinhardtii* v5.5 (ID: Cre02.g073650) and isolated by RT-PCR. The homologous sequences of CrPrp19 were obtained using BLASTp in the NCBI database.^1^ Multiple sequence alignments were performed using the ClustalW program.^2^ Neighbor-joining (NJ) phylogenetic tree was constructed by MEGA7.0 based on the JTT model, using a bootstrap value of 1,000 as default parameters (Kumar et al., 2016). Compute pI/Mw tool^3^ was used to calculate molecular weight and p*I* value of CrPrp19. Motifs were annotated by the conserved domain database using the SMART program.^4^ Subcellular localization was predicted with Cell-PLoc 2.0.^5^ Transmembrane regions and orientation were predicted by TMHMM program.^6^ Comparative 3D protein prediction was obtained using Phyre2.^7^

### Expression Profile of *CrPrp19* Gene

Total RNA was extracted using Trizol reagent (Invitrogen, Carlsbad, CA, United States) following the manufacturer’s instructions. About 0.1 mg of total RNA was reversed to the first cDNA using the PrimerScript RT Reagent Kit with gDNA Eraser (Takara, Beautilion, Otsu, Japan). Primers used for qRT-PCR are listed in Supplementary Table 1. The quantified expression level of the target gene was normalized against the housekeeping gene, *18S rRNA* (GenBank accession no. MF101220.1). ABI-7500 Fast Real-Time PCR System (Applied Biosystems, Foster City, CA, United States) was applied for qRT-PCR using the SYBR Green master mix (Takara). The conditions for quantitative analysis were as follows: 94°C for 2 min; 35 cycles of 94°C for 10 s, 62°C for 15 s, and 72°C for 20 s; and a final extension at 72°C for 10 min. Gene expression was normalized with the 2^–ΔΔ*Ct*^ method setting HSM-cultured groups as controls.

### Vector Construction and Transformation

Plasmid p*Maa7* IR/ *CrPrp19* IR, which was used for knockdown of *CrPrp19* in *C. reinhardtii*, was constructed as follows. The primers that were used in this experiment are listed in Supplementary Table 1, and a fragment 278 bp from 5’-UTR to non-conservative domain-encoding region was amplified from cDNA by PCR and cloned into pMD18-T vector. After being digested by KpnI/BamHI and HindIII/SalI, the fragment was subsequently inserted into a modified vector pMD18T-*18S* as previously described by Deng et al. (2011). Finally, *CrPrp19* IR was double digested with KpnI and HindIII and inserted to p*Maa7* IR with EcoRI blunt-end, while overexpression plasmid pCAMBIA1302-*CrPrp19* was constructed by restriction digestion and ligation method using other primer pairs, which are also listed in Supplementary Table 1. For RNAi experiment, the plasmid was introduced into *C. reinhardtii* by the glass beads method (Kindle, 1990). Transformants were screened in TAP medium with 1.5 mM L-tryptophan, 5 mg/L paromomycin, and 5 μM 5-FI (Sigma-Aldrich, San Francisco, CA, United States). To obtain overexpression lines, the recombinant plasmid was transformed into cells *via Agrobacterium* meditation method and putative transformants were screened using a TAP medium, which was supplemented with 50 mg/L kanamycin. Finally, transgenic algae lines were confirmed by the transcription level of *CrPrp19* using qRT-PCR.

### Measurement of Cell Growth

Growth of the *C. reinhardtii CrPrp19* RNAi, *Maa7*, overexpression lines, and wild-type CC124 were determined by a Coulter Multisizer 4 (Beckman Coulter, Fullerton, CA, United States) daily. Cell sizes were measured using a microscope after synchronization culture referred elsewhere (Dillard et al., 2011). Moreover, cell responses to stresses such as -N, -S, and high salinity (8 g/L sodium acetate) were observed using the plated method.

### Nile Red Fluorescence Determination of Neutral Lipid Content

Neutral lipid (NL) was measured based on fluorescence intensities of 7-day-old cells staining with Nile Red (Sigma-Aldrich) according to a modified method reported by Chen et al. (2009). Liquid cell cultures were mixed with Nile Red and DMSO with a final concentration of 5 μg/ml and 20% (v/v), respectively. After being incubated in darkness for 15 min, mixtures were directly measured by a Glomax-Multi Detection System (Promega, Madison, WI, United States), with excitation and emission wavelengths of 530 and 575 nm, respectively. The standard curve between the concentration of neutral lipid (μg/ml) and the Nile Red fluorescence level was made based on using Triolein (Sigma-Aldrich) as a reference standard. NL contents (μg/10^6^ cells) were calculated using the following formula: NL(g/106*cells*) = cell0004×*FI*(530/575)−0.0038]×50/*celldensity*. For lipid drop observation, cells were cultured for 7 days and stained with Nile Red dye, then observed with a Nikon Eclipse 80i fluorescence microscope (Nikon, Tokyo, Japan) using an excitation and emission wavelength of 480 and 560 nm, respectively.

### Fatty Acid Methyl Ester Transformation and Fatty Acid Methyl Ester Analysis

For fatty acid methyl ester (FAME) transformation, 5–10 mg weighed freeze-dried cell powder was used. Cells were suspended in 1 ml NaOH–CH_3_OH solution (2 mol/L) and incubated at 75°C for 30 min with shaking. Methyl non-adecanoate (C15:0, 200 μg/ml; Sigma-Aldrich) was applied as the internal standard. After cool-down, 1 ml of HCl–CH_3_OH (4 mol/L) was added, and pH was adjusted to below 2.0 using HCl, and the mixture was then incubated at 75°C for 30 min with shaking. The methyl esters of FAs were extracted with 2 ml hexane, then dried by nitrogen blowing, and dissolved in 10 μl of CH_2_Cl_2_ for GC/MASS analysis. FAMEs were examined by Thermo Trace GC Ultra gas chromatograph coupled to Thermo Polaris Q mass spectrometry (Agilent, Santa Clara, CA, United States) equipped with an HP-5MS column (30 mm × 0.25 mm, film thickness 0.25 μm) as previously described (Wang et al., 2018).

### Yeast Two-Hybrid Analysis

A yeast two-hybrid assay was performed to find the binding proteins of CrPrp19 by using Matchmaker GAL4 Two-Hybrid Systems (Takara), following the manufacturer’s instructions. To construct bait vector pGBKT7-*CrPrp19*, CDS of *CrPrp19* was amplified using primers, which are listed in Supplementary Table 1 with the BamHI and PstI flanking restriction sites, then cloned through double digests and ligation. A total of 5 mg of *C. reinhardtii* total RNA was used to create the cDNA library. Both bait and cDNA library vectors were transformed together into yeast strain Y2H Gold and screened on quadruple dropout QDO/X/A medium (SD/-Ade/-His/-Leu/-Trp supplemented with X-a-Gal and Aureobasidin A). The plasmids of potential yeast positives were extracted and amplified in *E. coli*. All plasmids from *E. coli* were sequenced and blasted.

### Subcellular Localization of *CrPrp19*

For CrPrp19 localization assay, a GFP- CrPrp19 fusion protein expression vector was constructed and transformed into *C. reinhardtii* CC124 using *Agrobacterium*-mediated genetic transformation method. The coding region of *CrPrp19* cDNA without stop codon was amplified by using primer pairs, which are listed in Supplementary Table 1, then it was inserted into the NcoI and SpeI sites at the initiator codon downstream of the cauliflower mosaic virus (*CaMV*) 35S promoter in the plant cell expression vector pCAMBIA1302. The control plasmid, pCAMBIA1302, and plasmids containing the fused gene construct were transformed to *Agrobacterium* LBA4404, which were then co-incubated with cultured CC124 cells. After dark-repairing culture for 24 h at 24°C, cells were plated on TAP mediums with 50 mg/L kanamycin and 15 mg/L rifampin. The clones were imaged with the Nikon Eclipse 80i fluorescence microscope with an excitation wavelength of 488 nm and an emission wavelength of 530 nm.

### Statistical Analysis

Statistical analysis was performed using SPSS 11.5 program, and differences were analyzed with one-way ANOVA followed by Duncan’s multiple comparison test. Data are shown as mean ± SD from three independent experiments unless specified otherwise.

## Results

### Identification of *CrPrp19* Gene and Bioinformatics Analysis

**FIGURE 1:**
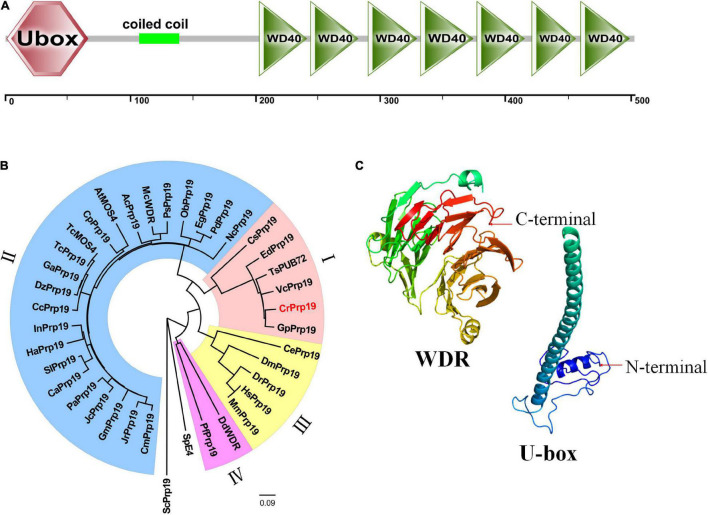
Features of CrPrp19 protein. **(A)** Scheme shows the domains of CrPrp19 protein. **(B)** NJ phylogenetic tree of CrPrp19 protein. The tree was constructed with the Mega7.0 with amino acid sequences of CrPrp19 homologous proteins retrieved by BLASTp searching in NCBI (GenBank accession numbers are listed in Supplementary Table 2). **(C)** Three-dimensional structure of U-box and WDR domain of CrPrp19.

The cDNA of *CrPrp19* comprises an ORF of 1,512 bp encoding a Prp19-like protein of 503 aa with a calculated molecular weight of 53.95 kDa and an isoelectric point of 6.61. CrPrp19 protein contains a conserved U-box at the N terminal, a coiled coil motif, and a WDR domain with seven WD repeats at C terminal (Figure 1A). None of the transmembrane helices was found in CrPrp19 according to TMHMM program. A total of 37 CrPrp19 homologous protein sequences were retrieved by BLASTp from the NCBI database. After multiple alignments, a Neighbor-Joining phylogenetic tree of CrPrp19 was constructed with bootstrap values of 1,000 replicates (Figure 1B). The phylogenetic analysis showed that the overall amino acid residues of Prp19 proteins had a relatively low conservation from yeast to human. Prp19 proteins from eukaryotes were divided into four major clades, microalgae (clade I), higher plants (clade II), animals (clade III), and fungi (clade IV). CrPrp19 was mostly closely related to GpPrp19 from *Gonium pectoral*, which was grouped with microalgae Prp19 proteins. Rather, Prp19 proteins from yeast formed a separate branch, showing a distinct generation relationship (Figure 1B). The three-dimensional protein prediction by Phyre 2 (Kelley et al., 2015) showed that CrPrp19 was structurally homologous with pre-mRNA-processing factor 19 of the yeast spliceosome at 3.6 Å resolution (PDB ID:c3jb9U_1), with a confidence of 100% (Figure 1C).

### Expression Patterns of *CrPrp19* Under Nutrition Deprivation

**FIGURE 2:**
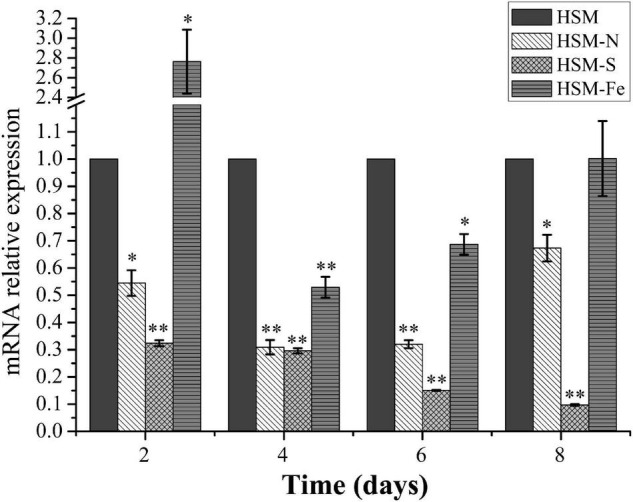
Effects of the deprivation of N, S, and Fe on the expression of *CrPrp19*. *18S rRNA* was used as an internal control. Expression was normalized to that of untreated HSM groups. Data are means ± SD (*n* = 3). Asterisks indicate significant differences at *p* ≤ 0.05 (*) or *p* ≤ 0.01 (**) compared with its control by one-way ANOVA.

Nutrition deprivation has been generally considered as an effective method to induce lipid biosynthesis in microalgae. To evaluate the expression profile of *CrPrp19* gene under nutrition deprivation conditions, *C. reinhardtii* cells cultured under nitrogen (N), sulfur (S), and iron (Fe) deficiencies stress for 8 days were collected for RNA isolation, and the untreated cells (HSM) were used as control. Gene expression of *CrPrp19* fold changes at the starvations were normalized with controls. As shown in Figure 2, mRNA expressions of *CrPrp19* were detected in all cells under treatments. However, compared with the controls, a significant decrease in *CrPrp19* gene expression was found under N and S deficiency stresses. More specifically, the transcript level of *CrPrp19* was remarkably decreased under N deficiency since 2 days and reached its lowest point at 4 days, which was approximately 30.9% lower than that of control. Meanwhile, the expression level of *CrPrp19* under S deprivation showed a similar expression pattern, which was dramatically reduced by S deficiency after 2 days, and the inhibition effect was on until 8 days, with a minimum transcript, 9.7% of control. Unlike N and S deprivation, the expression of *CrPrp19* was induced by deprivation of Fe at 2 days, but was markedly repressed after 4 and 6 days, which decreased by 47.1 and 31.3%, respectively (Figure 2). Taken together, the transcript of *CrPrp19* is deduced by nutrition deprivation, implying that it may be a regulator in response and adaptation to these nutrition stresses.

### Construction and Identification of *CrPrp19* RNA Interference and Overexpression Lines

**FIGURE 3:**
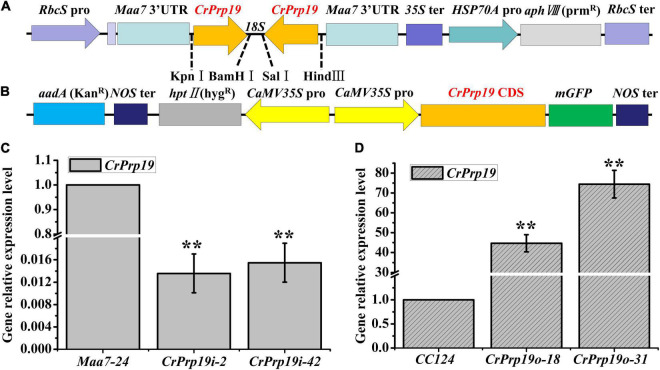
Schematic illustration of *CrPrp19* RNA interference and overexpression constructs and presentation of the resulting gene relative expression levels after transformation. **(A)** Schematic diagram of p*Maa7 IR*/*CrPrp19* IR drawn according to Deng et al. (2011). **(B)** Schematic diagram of pCAMBIA1302/*CrPrp19*. **(C)** Gene relative expression levels of two *CrPrp19* RNA silencing individuals during standard growth conditions; the values were normalized to the expression level of the empty vector p*Maa7* IR transgenic line. **(D)** Gene relative expression level of *CrPrp19* overexpression cell lines normalized to the expression level of the wild type; error bars indicate a standard error, asterisks (**) show significant differences at *p* < 0.01 compared with controls by one-way ANOVA.

To investigate the roles of *CrPrp19* gene in *C. reinhardtii* cells, RNA interference (RNAi) silencing and gene overexpression were carried out, respectively. A 278-bp fragment of *CrPrp19* cDNA was subcloned and inserted to p*Maa7* IR vector, generating the *CrPrp19* gene RNAi construct p*Maa7* IR/*CrPrp19* IR (Figure 3A). The coding region of *CrPrp19* was also cloned by RT-PCR and used to construct the gene overexpression binary vector pCAMBIA1302/*CrPrp19* (Figure 3B). After transforming p*Maa7* IR/*CrPrp19* IR and pCAMBIA1302/*CrPrp19* constructs into *C. reinhardtii* CC425 and CC124, respectively, antibiotic resistance transformants were generated. p*Maa7* IR vector was also transformed to *C. reinhardtii* CC425 as controls for RNAi experiment, and named as *Maa7* lines. After twice plating on selected plates, the survival transformants were randomly chosen for *CrPrp19* gene expression analysis by qRT-PCR. Two RNAi and two overexpression lines with the biggest differences in gene expression level compared with controls were selected, and named as *CrPrp19*i-2, *CrPrp19*i-42 and *CrPrp19*o-18, *CrPrp19*o-31, respectively. As shown in Figures 3C,D, *CrPrp19* mRNA abundance was decreased by 98.64 and 98.45% in RNAi lines, *CrPrp19*i-2 and *CrPrp19*i-42, respectively, while it increased by 44.67 and 74.42 times in overexpression lines, *CrPrp19*o-18 and *CrPrp19*o-31, respectively, compared with controls. The results suggested the high effectiveness of *CrPrp19* silencing and overexpression was achieved.

### *CrPrp19* Regulates Cell Growth and Neutral Lipids Accumulation in *Chlamydomonas reinhardtii*

**FIGURE 4:**
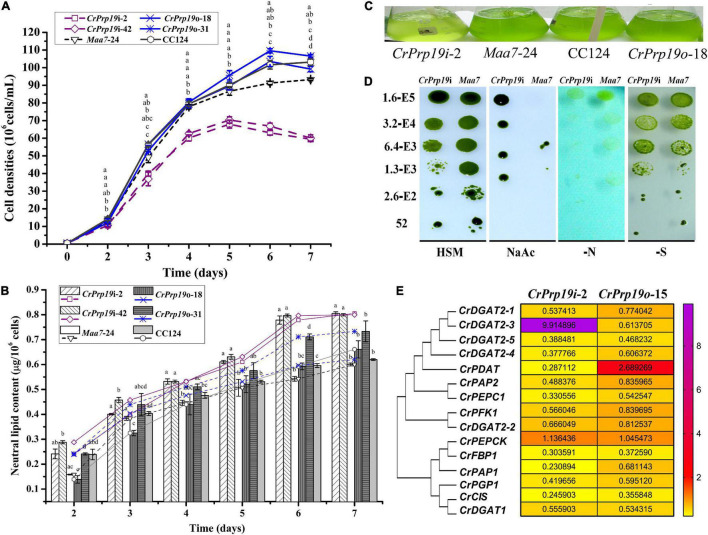
*CrPrp19* gene expression, cell growth, and NL contents of RNAi and overexpression transformants and their controls. NL contents were estimated by dividing cell density with NL concentration calculated by Nile Red fluorescent dye staining method. **(A)** Cell densities of *CrPrp19* RNAi and overexpression lines and controls. The different letters above each point indicate that they were significantly different at *p* ≤ 0.05 according to one-way ANOVA. **(B)** NL contents of *CrPrp19* RNAi and overexpression lines and controls. The different letters of each column indicate that they were significantly different at *p* ≤ 0.05 according to one-way ANOVA. **(C)** Aerated cultures of *CrPrp19* RNAi and overexpression transformants and their controls. **(D)** *CrPrp19* RNAi line (*CrPrp19*i-2) and *Maa7* line grown in stress plates. **(E)** Heat map of mRNA expression levels of lipid synthesis–related genes in *CrPrp19* RNAi and overexpression transformants, which was analyzed *via* qRT-PCR normalized with *Maa7* line and CC124, respectively.

To determine the function of CrPrp19 in cell growth and neutral lipid (NL) metabolism, *CrPrp19* silencing and overexpression lines were cultured in HSM medium for 7 days with the same initial cell density, and then the cell densities and NL contents were examined. The time-course profiles of cell growth and NL accumulation of different transgenic lines are shown in Figure 4. Compared with control *Maa7*-24 line, the cell densities of *CrPrp19* RNA interference lines were decreased significantly in *Maa7*-24 line as compared with control after cultured for 4 days (*p* < 0.01). However, cell growth had no discernible difference between *CrPrp19* overexpression line and wild-type strain, *p* > 0.05 (Figure 4A). The growth curves also showed that *CrPrp19* RNAi lines had a maximum cell concentration at day 5 with the highest values of 6.8 × 10^7^ and 7.0 × 10^7^ cells/ml. They were 21.5 and 19.2% less than that of the control group, respectively. Figure 4B shows that the contents of NL (μg/10^6^ cells) of two *CrPrp19* RNAi lines were more than that of controls since cultured for 4 days. After being cultured for 6 days, NL contents of *CrPrp19* RNAi lines became stable, which had a similar value of about 0.8 μg/10^6^ cells, which were 1.46 and 1.33 times more than that of the control line at day 6 and day 7. In contrast, overexpressed *CrPrp19* gene seems unregulated on NL accumulation in *C. reinhardtii*. *CrPrp19*o-18 line had less NL at day 2 and day 3 than CC124 strain, while lipid contents of *CrPrp19*o-31 lines were increased by 119% than that of control at 6 days. These results suggested that the silencing of *CrPrp19* gene could increase lipid accumulation, but this effect was at the price of less cell densities.

*CrPrp19* RNAi silencing and overexpressed lines and their respective controls were aerated cultured for 4 days, and the result showed that CrPrp19 RNAi silencing seemed more yellow than others (Figure 4C). Because of the growth difference between *CrPrp19* RNAi and *Maa7* line, a further comparison and analysis between the growth of *CrPrp19* RNAi and *Maa7* line under stresses conditions were performed. Here, a high concentration of sodium acetate (NaAc, 8 g/L) was also applied. Acetate is the carbon source usually used in heterotrophic cultivation to promote biomass of *C. reinhardtii*, but high concentration of NaAc inhibits *C. reinhardtii* metabolism. Appropriate amount of NaAc could also increase the yield of intracellular FA in *C. reinhardtii* mutant (Ramanan et al., 2013). The results showed that *CrPrp19* RNAi and *Maa7* line showed no obvious difference under nitrogen or sulfur deficiency, but much better tolerability to high concentration of NaAc than control (Figure 4D). The growth abilities of *CrPrp19* RNAi lines under high concentration of NaAc could be applied to produce lipids with NaAc addition.

Moreover, to reveal the changes of lipid synthesis–related genes in *CrPrp19* RNAi and overexpressed lines, qRT-PCR was applied. *CrDGAT2-3* was induced to be expressed in *CrPrp19* RNAi line, while the expression of *CrPDAT* increased in *CrPrp19* overexpressed line (Figure 4E). The transcript of *CrPEPCK* gene was stable in both *CrPrp19* RNAi and overexpressed lines, but expressions of other chosen lipid biosynthesis–related genes decreased. In summary, when *CrPrp19* was silenced or overexpressed, the majority of transcriptions of genes participating in lipid synthesis were changed, suggesting that *CrPrp19* could regulate lipid synthesis through control gene transcription.

**FIGURE 5:**
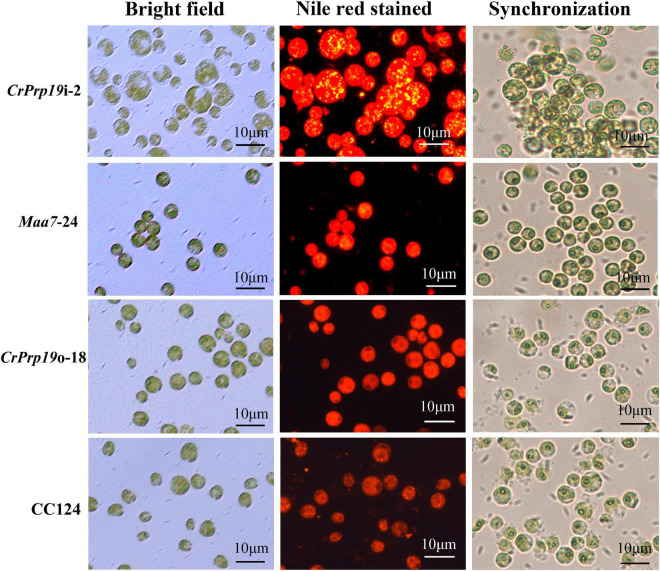
Cells and accumulated lipid droplets in *CrPrp19* RNAi and overexpression lines. Cells cultured for 7 days were stained by Nile Red staining and photographed by fluorescence microscopy. Scale bar, 10 μM. 1st row: Bright field, 2nd row: fluorescence microscopy images stained with Nile Red, red showed chlorophyll autofluorescence, yellow showed lipid drops. 3rd row: Cells after synchronization culture for 4 days.

The accumulation of lipid bodies in *CrPrp19* RNAi and overexpressed transformants was examined by fluorescence microscopy (Figure 5). After Nile Red staining, numerous fluorescent lipid droplets (LDs) were observed in *CrPrp19* RNAi cells, but none or only a few were present in control cells and overexpressed lines (Figure 5). *CrPrp19* RNAi cells were almost twice bigger than the control *Maa7*-24 line, while overexpression of *CrPrp19* did not change cell morphology. To verify this phenomenon, all microalgae strains were synchronized with 5% CO_2_ aerated cultured under long-day station. After synchronization culture, the *CrPrp19* RNAi cell lines also seemed larger than others (Figure 5), indicating that *CrPrp19* plays a critical role in cell size control.

### Fatty Acid Composition Analysis of *CrPrp19* RNAi and Overexpression Transformants

**FIGURE 6:**
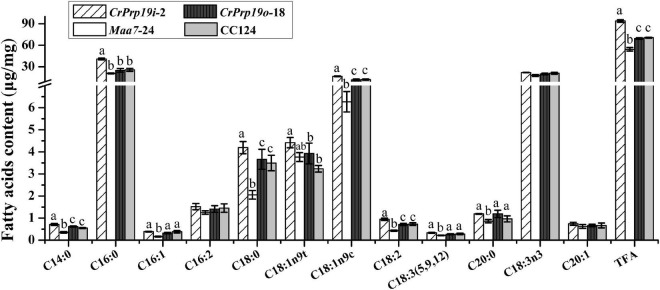
Fatty acid composition in *CrPrp19* RNAi and overexpression lines. FAMEs in transformant *CrPrp19*i-2, *CrPrp19*o-18, and controls were analyzed using GC-MS. TFA is total fatty acids. Each value represents mean ± SD (*n* = 3). Significant difference between transformant *CrPrp19*i-2, *CrPrp19*o-18, and controls by one-way ANOVA is indicated with different letters.

In general, the composition of FAs determined the quality of natural edible oils and biofuels. Therefore, we analyzed the FA composition of *CrPrp19* RNAi and overexpressed transformants. After transesterification, FAMEs were examined using GC-MS with methyl non-adecanoate (C15:0) as the internal standard. As shown in Figure 6, FAMEs of *CrPrp19* overexpressed line had no difference with the wild-type CC124, with *p* > 0.05, which was similar to the contents of total lipids (Figure 4B). Total FAMEs in *CrPrp19* RNAi showed a significant increase compared with control *Maa7*-24 line and wild-type CC124. Among FAs in microalgae lines, the highest contents were C16:0 and C18:3n3. The C16:0 in the *CrPrp19* RNAi line compared with Maa7 lines and wild type was increased 1.98 and 1.59 times, respectively, whereas there was no obvious distinction among *CrPrp19* RNAi lines and controls in C18:3n3 contents. Another high-content FA in *CrPrp19* RNAi transformants was C18:1n9c, which was 2.65 and 1.37 times more than that of *Maa7* lines and wild type. Others, such as C14:0 and C18:2 in *CrPrp19* RNAi lines, also showed significant accumulation than controls, with 2.02- and 2.20-fold increase. Besides, C16:1, C18:0, C18:3 (5, 9, 12), and C20:0 of *CrPrp19* RNAi lines were much more than that of *Maa7* lines. C18:1n9t in transformant *CrPrp19*i-2 compared with wild-type CC124 was increased by 36.6%, but the same as the *Maa7* line. The other FAs showed no significant difference. CrPrp19 may negatively regulate FA synthesis.

### Nuclear Localization of *CrPrp19*

**FIGURE 7:**
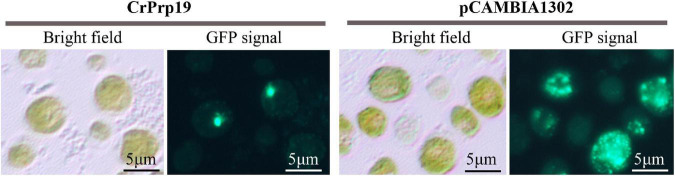
Nuclear localization of the CrPrp19 protein. *C. reinhardtii* CC124 cells were transformed *via Agrobacterium*-mediated genetic transformation method with a fusion gene construct including a truncated *CrPrp19* gene fused in frame with a *GFP* coding gene and driven by the *CaMV35S* promoter. Cells were fixed and observed by fluorescent microscopy using an excitation wavelength at 488 nm.

Protein subcellular localization predicted by Cell-PLoc 2.0 showed that CrPrp19 was a nuclear protein. To verify this result, a fusion construct containing the CrPrp19 gene fused in-frame with the GFP gene and driven by the *CaMV 35S* promoter was used to transform *C. reinhardtii* CC124 cells by *Agrobacterium*-mediated genetic transformation method. As shown in Figure 7, the cells of *C reinhardtii* CC124 successfully transformed with the chimeric *CrPrp19*-GFP gene exhibited GFP fluorescence only in the nucleus. Transformation of *C. reinhardtii* CC124 cells with the empty construct pCAMBIA1302, which contains *CaMV35S*/GFP gene construct but lacks the *CrPrp19* coding sequence, showed no evidence for nuclear localization of GFP activity. That is, in pCAMBIA1302, transformed cells of *C. reinhardtii* showing GFP were uniformly distributed throughout the cytoplasm of each cell. So CrPrp19 is a nucleoprotein.

### Interacting Protein of *CrPrp19*

Generally, a well understanding of protein interactions could help reveal the cellular functions of target proteins. Here, the yeast two-hybrid (Y2H) assay was conducted to screen interactions of CrPrp19 protein. The full-length coding *CrPrp19* gene was cloned in frame with GAL4 DNA-binding domain, and *C. reinhardtii* cDNA library was cloned with GAL4 activation domain. After detection of non-autoactivation of CrPrp19, the Y2H assays were performed according to the manufacturer of the yeast two-hybrid assay kit. Finally, 52 positive clones grown in SD/QDO/X-α-Gal/AbA (selects for activation of auxotrophic, chromogenic, and antibiotic resistance markers through positive interaction) medium were selected. The gene sequences were identified by PCR amplification, sequencing, and BLASTp analysis. After removing the repeat and invalid sequences, 12 proteins were chosen for gene cloning. Since some of these proteins had long coding genes, only gene fragments were cloned and sequenced from first time Y2H assays. Target genes were cloned into pGADT7 vector and interacted with CrPrp19 bait protein. Finally, six binding proteins were identified, and their information is listed in Table 1, i.e., structural maintenance of chromosomes (SMC) protein, growth factor receptor domain containing protein, peroxisomal coenzyme A diphosphatase, peroxisomal membrane protein 2, phosphoserine transaminase, and pyrophosphate phosphohydrolase.

**TABLE 1:** List of coding genes of putative interacting proteins of CrPrp19 identified by yeast two-hybrid assay.

No.	Gene symbol	Gene locus	Chromosome position*^a^*	Amino acids	Description
1	SMC	Cre06.g307250.t1.2	6:8434625..8442485R	1,286	Structural maintenance of chromosomes (SMC) protein
2	Hypothetical protein	Cre16.g668700.t1.2	16:4199135..4206852F	831	Hypothetical protein contains growth factor receptor domain
3	NUDT7	Cre02.g081300.t1.1	2:1115202..1119675R	737	Peroxisomal coenzyme A diphosphatase
4	PXMP2	Cre16.g668900.t1.1	16:4168064..4170253R	167	Peroxisomal membrane protein 2
5	PSAT	Cre07.g331550.t1.2	7:2791510..2794528R	406	Phosphoserine transaminase
6	PPase	Cre09.g387875.t1.1	9:3253978..3256301F	192	Pyrophosphate phosphohydrolase

*^a^F and R represent the forward and reverse directions on the chromosome, respectively.*

## Discussion

Prp19 complex, also named as NineTeen Complex (NTC), functions in several critical processes in eukaryotes, including RNA splicing, transcription elongation, genome maintenance, and protein ubiquitination degradation by the proteasome (Chanarat and Sträßer, 2013). As a large protein complex, NTC/Prp19C consists of eight core proteins and more than 30 associated proteins in higher eukaryotes. Although NJ tree in this study also demonstrated that PRP19 proteins from yeast to human had relatively low evolutionary homolog in complete sequences (Figure 1B), CrPrp19 showed high similarity with yeast Prp19 (Figure 1C), which has highly conserved residues essential for protein interactions and function (Kooi et al., 2010). Three recognized protein motifs, WD-40 repeat domain, U-box, and coiled-coil domain, were predicted in CrPrp19 (Figure 1A). These domains are essential for Prp19p tetramer structure formation, ubiquitination degradation, and interaction with other proteins. U-box domain of Prp19 forms dimer functioning as an E3 ligase mediating poly-ubiquitination (Kooi et al., 2006), and WDR structured a highly conserved surface centered around blade five that is required for the physical interaction (Kooi et al., 2010).

The most thoroughly examined role of NTC/Prp19C is splicing, which is a critical step in gene expression to remove introns (Yin et al., 2012). Prp19 proteins have vital physiological roles for regulation of gene expression networks. In *CrPrp19* mutants and overexpression lines, it was noticed that most of the genes that are related to synthesis of lipids decreased (Figure 4D). These findings indicated that CrPrp19 is important for mRNA maturation. Since ScPrp19 was identified in yeast, more and more members of this family have been explored. Duplication of Prp19 proteins was isolated in *Arabidopsis*, while a single copy of Prp19 was found in human and yeast, as well as in *C. reinhardtii*. CrPrp19 develop clusters with algae and plant Prp19 (Figure 1B), which most (showed resemblance with GpPrp19) likely reflects that plant Prp19 proteins have a common evolutionary origin. In plants, the scarce function of Prp19 was mostly found in stress tolerance and innate immunity (Monaghan et al., 2009; Zhang et al., 2017). CrPrp19 also responded to nutrition deficiencies, where its gene expressions were obviously altered under these conditions (Figure 2). CrPrp19 is possibly regulated by other proteins, which are involved in responding to environmental influences. About 95% of human and 61% of *Arabidopsis* intron-containing genes need to undergo alternative splicing, which is critical for growth and development, as well as for responses to changes in environmental conditions. As the core element of the spliceosome, Prp19 proteins are also involved in stress responses. This could explain why the expression of CrPrp19 gene modulated obviously under nutrition deprivation stresses. It requires further investigations to explain how Prp19 acts under different stresses to regulate cell development.

Among the core proteins of NTC, Prp19 proteins play key functions in many different cellular processes. Besides the roles of NTC, Prp19p has also been implicated in lipid droplet biogenesis of mouse adipocytes, which in *Prp19p* downregulated mouse pre-adipocyte cells, fewer lipid droplets were formed (Cho et al., 2007). However, this is inconsistent with our findings that more lipids were observed in the *CrPrp19* RNAi lines (Figures 4B, 5). One of the putative explanations may be because of the cellular distribution difference. In mouse adipocytes, Prp19p is located in both cellular and lipid droplets, but CrPrp19 was only detected in the nucleus (Figure 7), so these two kinds of Prp19 proteins function in lipid biosynthesis maybe *via* different pathways. Another explanation for RNAi silencing of *Prp19* from two species that resulted in distinct lipid body formation is that TAG biosynthesis of animals and plant has so many differences. In plants and animals, members from one protein family sometimes play various roles. For example, DGAT1 and DGAT2 proteins are positively functionally redundant for TAG accumulation in mice adipocytes under basal conditions, and DGAT2 is also essential in maintaining skin lipids (Chitraju et al., 2019); however, seven members of DGATs were found in *C. reinhardtii* having distinct roles during lipid biosynthesis (Liu et al., 2016). As described in a previous study, Prp19 protein controls TAG accumulation *via* indirectly regulating triacylglycerol synthesis enzymes, including DGAT-1 and GPAT (Cho et al., 2007). In this investigation, it has been found that the expression of CrDGAT2-3 gene in lipid-rich CrPrp19 RNAi lines significantly enhanced about 9.9-fold more than controls (Figure 4E), indicating that CrPrp19 could also regulate DGAT2-3 to modulate TAG biosynthesis. Since CrDGAT2-3 was not detected to interact with CrPrp19 by Y2H assay (Table 1), it suggested that the functions of CrPrp19 in lipid biosynthesis are independent of regulating CrDGAT2-3.

In microalgae, synthesis of neutral lipids uses glycerol-3-phosphate (G3P) as substrate. G3P is generated through the Calvin cycle and regulated by a series of carbon partitioning–related enzymes. Lysophosphatidic acid (LPA) is formed *via* G3P and acetyl-CoA acylation by glycerol-3-phosphate acyltransferase (GPAT) catalysis, and then LPA is generated as phosphatidic acid (PA) under LPAAT catalysis. DAG is synthetized by removing the phosphate group in the sn-3 position of PA catalyzed by PAP (Moellering et al., 2009), and finally TAG forms from DAGs. The enzymes modulating carbon flows in microalgae affect lipid accumulation too. Downregulated *CrPEPC1* gene increased biomass and lipid accumulation rate (Kao and Ng, 2017). It is a mystery that these enzymes, which are involved in DAG biosynthesis, displayed various degrees of decline when expression levels of CrPrp19 were changed (Figure 4E). As noted previously, plant peroxisomes, which are abundant in oil seeds along with lipid droplets, are important for plant TAG synthesis (Pracharoenwattana et al., 2005). Several peroxisome proteins were found to interact with CrPrp19, so speculation could be made that suppression of CrPrp19 resulted in the inhibition of ubiquitination degradation of peroxisome proteins mediated by CrPrp19 and subsequential lipid biosynthesis. The composition of fatty acids in microalgae determines the quality of lipids, especially their acceptability for use in biodiesel production. The higher amount of C16:0, C16:1, and C18:1n9c were detected in CrPrp19 RNAi lines (Figure 6), showing that FA composition of CrPrp19 RNAi lines was more fit for biodiesel synthesis.

However, overexpression of *CrPrp19* had little effects neither on content of lipids nor on cell phenotypes (Figures 4, 5). These data clearly support previous results from mouse Prp19p overexpression 3T3-L1 cells, in which overexpression of Prp19 also had no effect on TAG synthesis (Cho et al., 2007). This further indicates that Prp19 is not a stimulus for lipid storage in cells. Interestingly, the observed benefits of *CrPrp19* overexpression were the increased expression of PGAT1 (Figure 4E), which is a key enzyme responsible for TAG biosynthesis in microalgae; however, the increased degree was not enough to cause changes in lipid content.

Accumulation of high-value products, including lipid, was against cell growth, and previous studies showed that carbon availability was the major limiting factor for product biosynthesis and cell reproduction. High carbon/nitrogen ratio was capable of stimulating algal cells cycle under nitrogen starvation fed-batch culture condition (Sun et al., 2020). This study suggested that silencing of *CrPrp19* caused inhibition of cell cycles (less cell densities, Figure 4A) and abundance of lipid biosynthesis (Figure 4B), which would result from repression in chemical energy consumption by general metabolism that slows down, and the diversion of the chemical energy to conditionally induce metabolism, including TAG synthesis. Normally, lipid content in cell is more in late stationary phase than in logarithmic phase, when cell growth is repressed (Deshmukh et al., 2019). Similar metabolic changes would occur in KO lines that are repressed in cell division and growth. The well growth of *CrPrp19* mutant on NaAc medium (Figure 4D) is consistent with the previous reports that addition of NaAc (4 g/L) significantly increased biomass of *C. reinhardtii* under the absence of nitrogen absence condition (Yang et al., 2018). Cultured *CrPrp19* RNAi lines under high concentration of NaAc may be applied to produce high lipids and biomass.

Silencing of *CrPrp19* not only significantly increased the lipid contents but also enlarged cell sizes (Figure 5). As one member of spliceosome, Prp19-associated complex is essential for cell development through regulating intron splicing in several model species, including yeasts and human cells. The splicing factor Cwf15, a component of Prp19-associated complex, plays important roles in fungal growth, and the deletion of Cwf15 resulted in a shorter cell length (Liu et al., 2021). Previous studies showed that mutations in splicing factors could affect plant growth, development, and stress responses. The loss of the splicing factor PRP40C caused a late flowering phenotype in medium or long-day photoperiods, and a significant enrichment for genes related to salt and biotic stress responses (Hernando et al., 2019). However, CrPrp19 RNAi mutants showed larger cell sizes (Figure 5), indicating the different roles of CrPrp19 in plant cells. Prp19 might be necessary for the cell cycle, so inhibition of CrPrp19 resulted in decreased cell densities (Figure 4A). As we know, the quantities of cells depend on mitosis in single-celled organism *C. reinhardtii*. Human Prp19 is known to directly contribute to functions in open mitosis independent of its role in the interphase nucleus as the first spliceosome subcomplex (Hofmann et al., 2013). CrPrp19 interacts with SMC protein, which generally binds to DNA and is induced early in S phase, acting in organizing and segregating chromosomes for cell division. The RNAi of CrPrp19 may disrupt the normal cell cycle and cause abnormal cell division, which also resulted in cell sizes changes. During biodiesel production in microalgae, harvesting is one of the major energy and cost consumption progress, which is against the “green energy” concept. Generally, appropriate harvesting technique with low energy consumption and less cost maintenance majorly is selected depending on cell size and density (Vasistha et al., 2021). The larger cell size of *CrPrp19* RNAi lines makes it easier to be collected, but unfortunately, the cell densities decreased significantly (Figure 4A), which needs to be improved for direct applications.

PRP19 in humans and yeast often binds to Pre4/β7 subunit, which is the central component of 20S proteasome, while mouse PRP19 binds to the 19S proteasome regulatory subunit SUG1 (Löscher et al., 2005; Sihn et al., 2007). Yet, the interaction proteins of CrPrp19 in this study are not subunits of proteasomes, but some metabolic enzymes and cell cycle–related proteins (Table 1). This may account for the mechanism of CrPrp19 regulating cell growth and lipid biosynthesis. To date, Prp19p has countless repeated functions in nucleus, and we also found CrPrp19 located in the nucleus exclusively (Figure 7). Based on the phenotypes of *CrPrp19* RNAi lines and the protein–protein interactions analysis, we speculated that CrPrp19 might also act in the cell cycle control and regulate lipid formation *via* an indirect pathway. Nevertheless, detailed molecular mechanisms of CrPrp19 functions still need more studies to reveal.

In summary, according to our results, the achievement of microalgae with more lipids and larger cell size through *CrPrp19* RNA interference silencing can be applied as an alternative strategy for the development of microalgal strains that meet industrial biofuel production.

## Data Availability Statement

The original contributions presented in the study are included in the article/Supplementary Material, further inquiries can be directed to the corresponding author/s.

## Author Contributions

QL and YL performed the experiments. QL, HZ, and YL analyzed the data. CW and ZH provided reagents and materials. QL and XZ wrote the article. ZH revised the article. QL and ZH conceived and designed the experiments. All authors read and approved the final manuscript.

## Conflict of Interest

The authors declare that the research was conducted in the absence of any commercial or financial relationships that could be construed as a potential conflict of interest.

## Publisher’s Note

All claims expressed in this article are solely those of the authors and do not necessarily represent those of their affiliated organizations, or those of the publisher, the editors and the reviewers. Any product that may be evaluated in this article, or claim that may be made by its manufacturer, is not guaranteed or endorsed by the publisher.

## Footnotes

1
https://www.ncbi.nlm.nih.gov/

2
http://www.clustal.org/clustal2/

3
http://kr.expasy.org/tools/pi_tool.html

4
http://smart.embl-heidelberg.de/

5
http://www.csbio.sjtu.edu.cn/bioinf/Cell-PLoc-2/

6
http://www.cbs.dtu.dk/services/TMHMM/

7
http://www.sbg.bio.ic.ac.uk/phyre2/html
