# Supplementary material for: A U-Box Type E3 Ubiquitin Ligase Prp19-Like Protein Negatively Regulates Lipid Accumulation and Cell Size in Chlamydomonas reinhardtii
Source: Front Microbiol. 2022 Apr 6;13:860024. doi: 10.3389/fmicb.2022.860024 (PMC9019728; doi:10.3389/fmicb.2022.860024)
Supplement: Supplementary file 1 [file Table_1.DOCX]

**Supplementary Table 1.** Primers used in this experiment

| **Primers uses** | **Primer name** | **Primer sequences (5’ - 3’)** |
| --- | --- | --- |
| cDNA cloning and constructing *CrPrp19* expression vectors | *CrPrp19*-cDNA-clone-F | GTATTGTTGACGTCCTTGCC |
|  | *CrPrp19*-cDNA-clone-R | GATTACAGGCCCCGATGATG |
|  | *CrPrp19*-pCAMBIA1302-over-F | GGCCATGGATGTTTTGCTCTATCAGCGG |
|  | *CrPrp19*-pCAMBIA1302-over-R | CGCACTAGTTTAAGCGGAGGGGCTGGAGA |
|  | *CrPrp19*-pCAMBIA1302-sub-F | GGCCATGGATGTTTTGCTCTATCAGCGG |
|  | *CrPrp19*-pCAMBIA1302-sub-R | CGCACTAGTAGCGGAGGGGCTGGAGAACA |
|  | *CrPrp19*-pGBKT7-F | GCGGATCCATGTTTTGCTCTATCAGCGG |
|  | *CrPrp19*- pGBKT7-R | CGCCTGCAGTTAAGCGGAGGGGCTGGAGA |
|  | *CrPrp19*-pGEX-6p-1-F | GCGGATCCATGTTTTGCTCTATCAGCGG |
|  | *CrPrp19*-pGEX-6p-1-R | CGCCTCGAGTTAAGCGGAGGGGCTGGAGA |
| q RT-PCR assays | *CrPrp19*-qF | GGCAACTAATGCGCTTCAGA |
|  | *CrPrp19*-qR | TGGCTCGTTACGGTCAAGAT |
|  | *18S rRNA*-qF | TCAACTTTCGATGGTAGGATAGTG |
|  | *18S rRNA*-qR | CCGTGTCAGGATTGGGTAATTT |
|  | *CrPEPCK*-qF | TGGACGACCGCACCTTTCTG |
|  | *CrPEPCK*-qR | TCCTCCTCCGTGGGCTTGAT |
|  | *CrPGP1*-qF | TAGATGGCACGCTCTGGAAA |
|  | *CrPGP1*-qR | CTGATGGCTTTGAGGTAGGC |
|  | *CrPAP1*-qF | CCTTCATCATGCCCGCCTACT |
|  | *CrPAP1*-qR | ACGCCCAGGTAAAGCCGAGA |
|  | *CrPAP2*-qF | GCGTGTTTGCCTACTTCCTC |
|  | *CrPAP2*-qR | CACTACTCGCGCCGTACAT |
|  | *CrPDAT*-qF | GCCTGGCTTGACCAGACCCT |
|  | *CrPDAT*-qR | GCCGACTTGAGCACCTTGGA |
|  | *CrFBP1*-qF | TACGAGCCCAGCGAGGAGTG |
|  | *CrFBP1*-qR | GTCCAGCGTGAAGCCGAACA |
|  | *CrPFK1*-qF | CACCTACGCCGAGTTCCAGT |
|  | *CrPFK1*-qR | GCGGTGAACAATGCCCTGTA |
|  | *CrPEPC1*-qF | TTTTGGAGCCGTGAGGGACG |
|  | *CrPEPC1*-qR | CGATGCCGGACAGGTTGAGG |
|  | *CrCIS*-qF | TTCCACGAGGCTGTGATGC |
|  | *CrCIS*-qR | CCTTGCGACCCGTGCTTT |
|  | *CrDGAT2-1*-qF | GCCGCCTCCGTCGTGTTCTA |
|  | *CrDGAT2-1*-qR | ATGCCGTCCACCTGCTCCTC |
|  | *CrDGAT2-2*-qF | GACGGTGCAAGTGGCGATGA |
|  | *CrDGAT2-2*-qR | GGCGGTGGTAGTGGTGATGG |
|  | *CrDGAT2-3*-qF | ACGGAGAAGGGCAAGCAAAG |
|  | *CrDGAT2-3*-qR | TGGAAGATCCAGGAGCGACA |
|  | *CrDGAT2-4*-qF | TTCGGATTCTGCCCGCACTC |
|  | *CrDGAT2-4*-qR | CAACAGGCCGCTGATGCTCT |
|  | *CrDGAT2-5*-qF | CACGCTGGCTTCAAACTTCT |
|  | *CrDGAT2-5*-qR | CGCAGCACCAGGTCATAAGT |
|  | *CrDGAT1*-qF | ACAAGGACTGGTGGAATGCG |
|  | *CrDGAT1*-qR | ACAGCCGACACGAAGAACGT |
| Constructing *CrPrp19* RNA interference expression vector | *CrPrp19*-RNAi-F | CTTGAGGCATTCTGCGATCTTG |
|  | *CrPrp19*-RNAi-R | ATCCCACTCATCGTGGAACAGG |

F, forward; R, reverse
