# Supplementary material for: A U-Box Type E3 Ubiquitin Ligase Prp19-Like Protein Negatively Regulates Lipid Accumulation and Cell Size in Chlamydomonas reinhardtii
Source: Front Microbiol. 2022 Apr 6;13:860024. doi: 10.3389/fmicb.2022.860024 (PMC9019728; doi:10.3389/fmicb.2022.860024)
Supplement: Supplementary file 2 [file Table_2.DOCX]

**Supplementary Table 2.** Protein sequences used for the construction of phylogenetic tree

| **Protein Name** | **Species** | **GenBank Accession number** |
| --- | --- | --- |
| CrPrp19 | *Chlamydomonas reinhardtii* | XP_001701820.1 |
| AcPrp19 | *Aquilegia coerulea* | PIA36239.1 |
| CaPrp19 | *Capsicum annuum* | XP_016566047.1 |
| CpPrp19 | *Carica papaya* | XP_021899515.1 |
| CmPrp19 | *Castanea mollissima* | KAF3951740.1 |
| CsPrp19 | *Chlorella sorokiniana* | PRW20323.1 |
| CcPrp19 | *Corchorus capsularis* | OMO86569.1 |
| DzPrp19 | *Durio zibethinus* | XP_022760346.1 |
| EdPrp19 | *Edaphochlamys debaryana* | KAG2485908.1 |
| EgPrp19 | *Elaeis guineensis* | XP_010920296.1 |
| GpPrp19 | *Gonium pectorale* | KXZ54344.1 |
| GaPrp19 | *Gossypium arboreum* | XP_017611730.1 |
| SpE4 | *Schizosaccharomyces pombe* | NP_594874.1 |
| ScPrp19 | *Saccharomyces cerevisiae S288C* | NP_013064.1 |
| PfPrp19 | *Plasmodium falciparum 3D7* | XP_001351160.1 |
| MmPrp19 | *Mus musculus* | NP_598890.1 |
| HsPrp19 | *Homo sapiens* | NP_055317.1 |
| GmPrp19 | *Glycine max* | XP_003555746.1 |
| DmPrp19 | *Drosophila melanogaster* | NP_523783.1 |
| DdWDR | *Dictyostelium discoideum AX4* | XP_642890.1 |
| DrPrp19 | *Danio rerio* | NP_958875.1 |
| CePrp19 | *Caenorhabditis elegans* | NP_001380015.1 |
| AtMOS4 | *Arabidopsis thaliana* | NP_850206.4 |
| VcPrp19 | *Volvox carteri f. nagariensis* | XP_002948553.1 |
| TcMOS4 | *Theobroma cacao* | EOY06172.1 |
| TsPUB72 | *Tetrabaena socialis* | PNH07333.1 |
| SlPrp19 | *Solanum lycopersicum* | XP_004247768.1 |
| PaPrp19 | *Populus alba* | XP_034926297.1 |
| PdPrp19 | *Phoenix dactylifera* | XP_038973040.1 |
| PsPrp19 | *Papaver somniferum* | XP_026422555.1 |
| ObPrp19 | *Oryza brachyantha* | XP_006661845.2 |
| HaPrp19 | *Helianthus annuus* | XP_021981928.1 |
| InPrp19 | *Ipomoea nil* | XP_019180544.1 |
| JcPrp19 | *Jatropha curcas* | XP_012077597.1 |
| JrPrp19 | *Juglans regia* | XP_035548061.1 |
| McWDR | *Macleaya cordata* | OVA02324.1 |
| NcPrp19 | *Nymphaea colorata* | XP_031483715.1 |
| TcPrp19 | *Theobroma cacao* | XP_017975021.1 |
